# A case of intestinal T‐cell lymphoma, not otherwise specified, that showed characteristic findings by magnified endoscopy combined with narrow‐band imaging

**DOI:** 10.1002/deo2.319

**Published:** 2023-11-27

**Authors:** Yuki Hirose, Satoshi Saito, Takanori Nishiguchi, Dai Yamazaki, Tsubasa Tateishi, Yuuichi Saito, Yukiko Komeno, Makoto Kodama, Shiho Iwamoto, Masayuki Fukata, Minako Sako

**Affiliations:** ^1^ Department of Gastroenterology Tokyo Yamate Medical Center Japan Community Healthcare Organization Tokyo Japan; ^2^ Inflammatory Bowel Disease Center Tokyo Yamate Medical Center Japan Community Healthcare Organization Tokyo Japan; ^3^ Department of Hematology Tokyo Yamate Medical Center Japan Community Healthcare Organization Tokyo Japan; ^4^ Department of Pathology Tokyo Yamate Medical Center Japan Community Healthcare Organization Tokyo Japan

**Keywords:** intestinal T‐cell lymphoma, lymphoproliferative disease, magnified endoscopy, narrow band imaging, tree‐like appearance

## Abstract

T‐cell lymphoma in the gastrointestinal tract (intestinal T‐cell lymphoma, [ITCL]) is rare. ITCL, not otherwise specified (ITCL, NOS) which is a type of ITCL is particularly rare. There are few case reports of ITCL, NOS but no previous reports describe its endoscopic features. In this report, the 69‐year‐old man was diagnosed with ITCL, NOS. Colonoscopy revealed the elevated legion and edematous mucosa with focal depressions in the lower rectum. On the depressed legions, magnifying endoscopy combined with narrow‐band imaging detected the disappearance of glandular structure and branching abnormal blood vessels like a tree. These findings were similar to the tree‐like appearance, which has been described as a unique feature of gastric mucosal‐associated lymphoid tissue lymphoma. The targeted biopsy of the tree‐like appearance showed abnormal histopathological findings which fit the definition of ITCL, NOS. He was treated with chemotherapy and achieved complete remission. As is the case of gastric mucosal‐associated lymphoid tissue lymphoma, the tree‐like appearance is possibly the unique sign of ITCL, NOS. We report the endoscopic features of ITCL, NOS and show characteristic findings by magnifying endoscopy combined with narrow‐band imaging.

## INTRODUCTION

Primary gastrointestinal (GI) lymphomas account for 1–8% of GI malignancies.^1^ Most primary GI lymphomas are B‐cell lymphomas. T‐cell types are very rare and account for 4–6% of primary GI lymphomas.^2^ Intestinal T‐cell lymphoma (ITCL) is the typical type of primary T‐cell lymphoma in the GI tract in the World Health Organization classification. The major types of ITCL are enteropathy‐associated T‐cell lymphoma (EATL) and monomorphic epitheliotropic intestinal T‐cell lymphoma (MEITL). ITCL, not otherwise specified (ITCL, NOS) is classified as not conforming to other ITCLs, anaplastic large cell lymphoma, or extranodal NK/T‐cell lymphoma.^3^ ITCL, NOS is extremely rare and lacks consensus on its endoscopic features. We describe a case of ITCL, NOS of the rectum, and present characteristic findings of magnifying endoscopy (ME) combined with narrow‐band imaging (NBI).

## CASE REPORT

**FIGURE 1:**
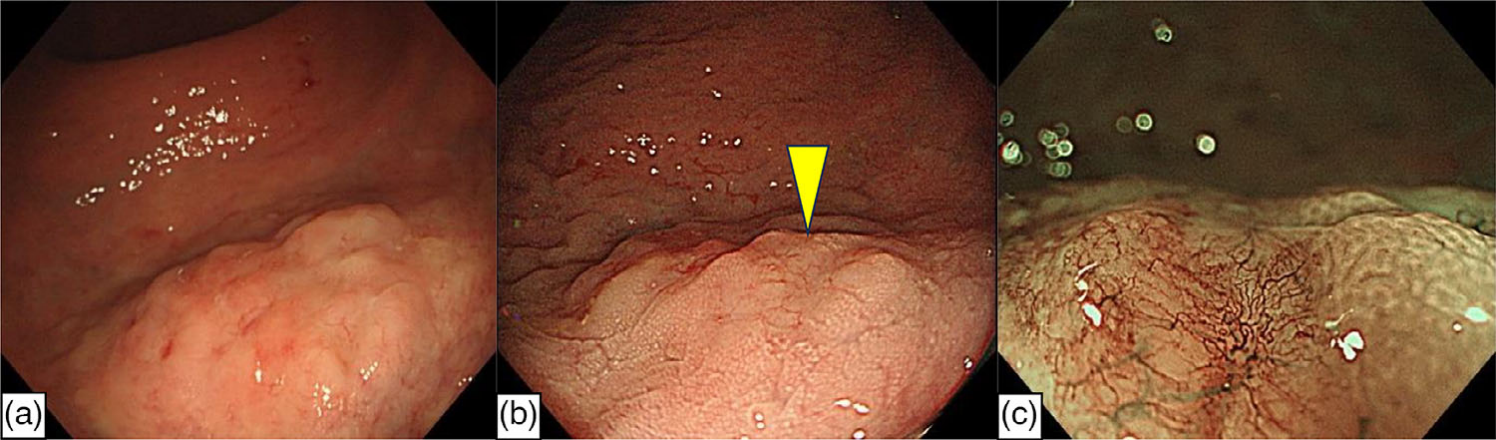
Endoscopic images in the lower rectum. (a) At the first examination, an elevated superficial lesion with a diameter of 20 mm was seen. Focal depressions were seen on the legion, but abnormal change of vascularity was unclear. (b) At the second examination, slight vascular dilatation was seen on the depressed legions (yellow arrowhead). (c) Narrow‐band imaging showed abnormal hypervascularity on the depressed lesions.

**FIGURE 2:**
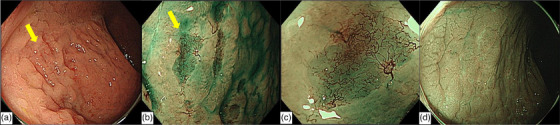
Endoscopic images in the lower rectum. (a) The edematous mucosa with focal depressions was distributed unilaterally (yellow arrow). (b) Narrow‐band imaging showed abnormal hypervascularity on the depressed lesions. (c) Magnifying endoscopy combined with NBI showed the disappearance of the glandular structure and the branching of abnormal blood vessels like a tree. (d) After chemotherapy, narrow‐band imaging showed that the depressed lesions became scarred and the abnormal hypervascularity disappeared.

**FIGURE 3:**
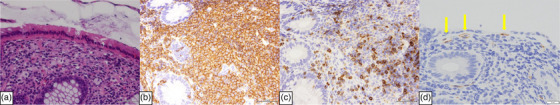
Histopathological examination of the rectal mucosa. (a) Small‐ to medium‐sized abnormal lymphocytes were infiltrated in lamina propria (hematoxylin and eosin stain, magnification: ×400). (b) These abnormal cells were positive for CD3 (CD3 stain, magnification: ×400). (c) These abnormal cells showed the downregulation of CD7 expression (CD7 stain, magnification: ×400). (d) Abnormal microvessels were observed immediately under the superficial layer of the mucosa and became flattened due to being compressed by infiltrated abnormal lymphocytes (yellow arrow, positive for CD31). The normal glandular structures were lost in lamina propria (CD31 stain, magnification: ×400).

**FIGURE 4:**
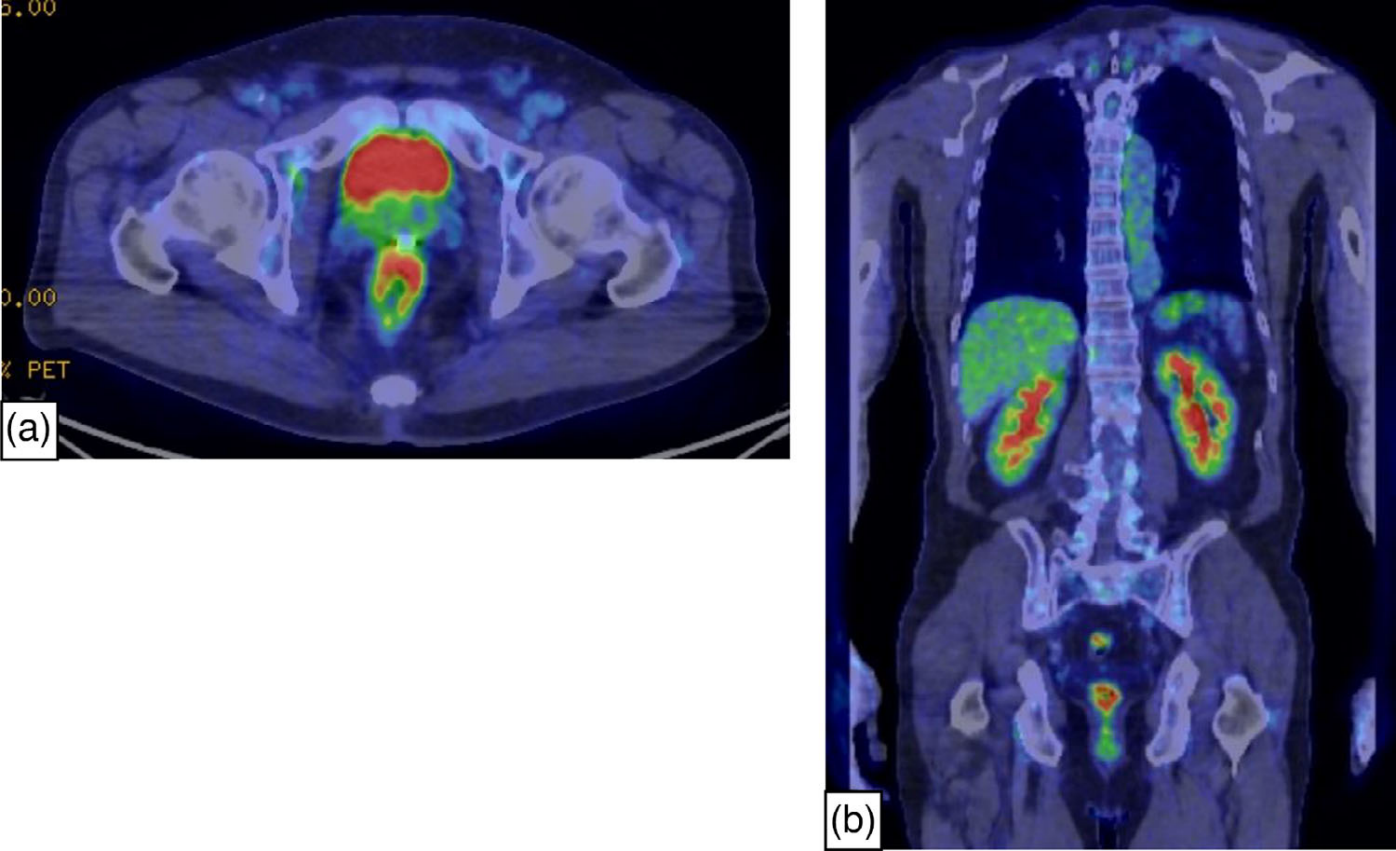
Endoscopic images in the lower rectum after chemotherapy. (a) The elevated lesion and edematous mucosa disappeared and the depressed lesions became scarred. (b) Narrow band imaging showed the disappearance of abnormal hypervascularity.

The patient was a 69‐year‐old man who underwent periodical follow‐up colonoscopies every year because of colon polyps. Colonoscopy revealed no abnormalities in the previous year. He had a history of pharyngeal and esophageal cancers, which had achieved a complete response to chemoradiation therapy 13 years ago. A colonoscopy was performed as usual without any complaint and revealed an elevated lesion with a diameter of 20 mm in the lower rectum (Figure 1a). The lesion was considered a neoplastic disease in the submucosa, but biopsy specimens showed no malignancies. He was referred to our department for re‐examination. Laboratory findings showed normal levels of lactate dehydrogenase (LDH 202 U/l) and soluble interleukin‐2 receptor (sIL‐2R 369 U/mL). Re‐examination colonoscopy revealed the elevated legion (Figure 1b) and edematous mucosa with focal depressions which were distributed unilaterally in the lower rectum (Figure 2a). NBI showed abnormal hypervascularity on the parts of depressed areas (Figures 1c and 2b). ME combined with NBI showed the disappearance of glandular structure and branching of abnormal blood vessels like a tree. These findings were consistent with the tree‐like appearance (TLA) described by Nonaka et al. (Figure 2c).^4^ We obtained biopsy specimens from the legions of TLA, and the histopathological examination revealed the infiltration of small‐ to medium‐sized abnormal lymphocytes in lamina propria (Figure 3a). Immunohistochemical examinations showed the expression of CD3 and the downregulation of CD7 expression, so these cells were mature T‐cell lymphoma (Figure 3b, c). Immunohistochemical examinations also demonstrated that these cells expressed CD2, CD4, CD5, and CD8 but not CD15, CD20, CD30, CD56, or granzyme B. These lymphoma cells were negative for the Epstein‐Barr encoding region (in situ hybridization). Positron emission tomography‐computed tomography showed the localized intense uptake of 18F‐fluorodeoxyglucose (FDG) at the lateral side of the rectum. The maximum standardized uptake value was 8.42. The FDG uptake showed more extensive legion compared to the area of abnormal mucosa detected by colonoscopy. Other parts of the body did not have an obvious abnormal uptake of FDG (Figure 4a,b). The rectal legion was not detected by contrast‐enhanced computed tomography. No other signs of lymphoma were found by esophagogastroduodenoscopy and contrast‐enhanced computed tomography. According to the above results, we made the final diagnosis of the present case as ITCL, NOS of the rectum. He received six cycles of chemotherapy with CHOP (cyclophosphamide, doxorubicin, vincristine, prednisone) and achieved complete remission (Figure 2d).

## DISCUSSION

ITCL is very rare, accounting for less than 5% of primary GI lymphomas.^5^ ITCL, NOS is extremely rare, so the clinical characteristics and endoscopic features have not been established. There is no established standard therapy for ITCL, but chemotherapy with CHOP has been used commonly. The median survival time of ITCL, NOS was reported about 35 months. ITCL, NOS have an aggressive course but better prognosis than EATL and MEITL.^3^ To the best of our knowledge, no previous studies on the endoscopic features of ITCL, NOS have been reported. Diffuse infiltrating type was reported as the characteristic endoscopic finding of T‐cell lymphomas such as MEITL and adult T‐cell leukemia/lymphoma.^6^ The present case showed elevated legion and edematous mucosa with focal depressions and was classified as diffuse infiltrating type. Microscopic features of ITCL, NOS are also not established because of the heterogeneity in morphology and immunophenotype. The present case was considered as ITCL because of the small‐ to medium‐sized abnormal lymphocytes which showed the expression of CD3, the downregulation of CD7 expression, and no expressions of CD 30 and CD56. These cells were positive for CD4 and CD5, which are usually negative in EATL and MEITL,^7^ so we diagnosed this case as ITCL, NOS.

ME combined with NBI demonstrated the TLA (in which abnormal blood vessels appear similar to the branches from the trunk of a tree) on the depressed legions in the lower rectum. This has been described as a unique feature that is suggestive of gastric mucosal‐associated lymphoid tissue lymphoma.^4^ The TLA has also been reported in rectal mucosal‐associated lymphoid tissue lymphoma^8^ and intestinal follicular lymphoma,^9^ but never in T‐cell lymphomas. Nonaka et al. described that the TLA reflects the loss of the glandular structure due to marked lymphoma cell infiltration and angiogenesis under the superficial layer of the mucosa.^4^ In our case, abnormal microvessels were observed immediately under the superficial layer of the mucosa and became flattened due to being compressed by infiltrated abnormal lymphocytes (Figure 3d). In this legion, the normal glandular structures were lost in lamina propria.

The present case was diagnosed by re‐examination of colonoscopy and histopathology. A correct diagnosis was made based on the examination of the biopsy specimens obtained from the site of the TLA, which had been detected by ME combined with NBI. Similar to a previous report related to gastric mucosal‐associated lymphoid tissue lymphoma, targeted biopsy of the TLA was useful in the diagnosis of lymphoproliferative diseases, including the present case.^10^

This report is the first to describe the endoscopic features of ITCL, NOS, and the relationship between the TLA and ITCL, NOS. This case suggests new information on the specific endoscopic features of ITCL, NOS and could contribute to the endoscopic diagnosis. Although our report is limited to a single case, the TLA could be a unique endoscopic feature that is suggestive of ITCL, NOS.

## CONFLICT OF INTEREST STATEMENT

None.

## Supporting information




FIGURE S1 Histopathological examination of the rectal mucosa at the site of the tree‐like appearance (CD31 stain, magnification: ×200)
