# Supplementary material for: A case of intestinal T‐cell lymphoma, not otherwise specified, that showed characteristic findings by magnified endoscopy combined with narrow‐band imaging
Source: DEN Open. 2023 Nov 27;4(1):e319. doi: 10.1002/deo2.319 (PMC10680999; doi:10.1002/deo2.319)
Supplement: Supplementary file 1 — FIGURE S1 Histopathological examination of the rectal mucosa at the site of the tree‐like appearance (CD31 stain, magnification: ×200) [file DEO2-4-e319-s001.pptx]

## Slide 1
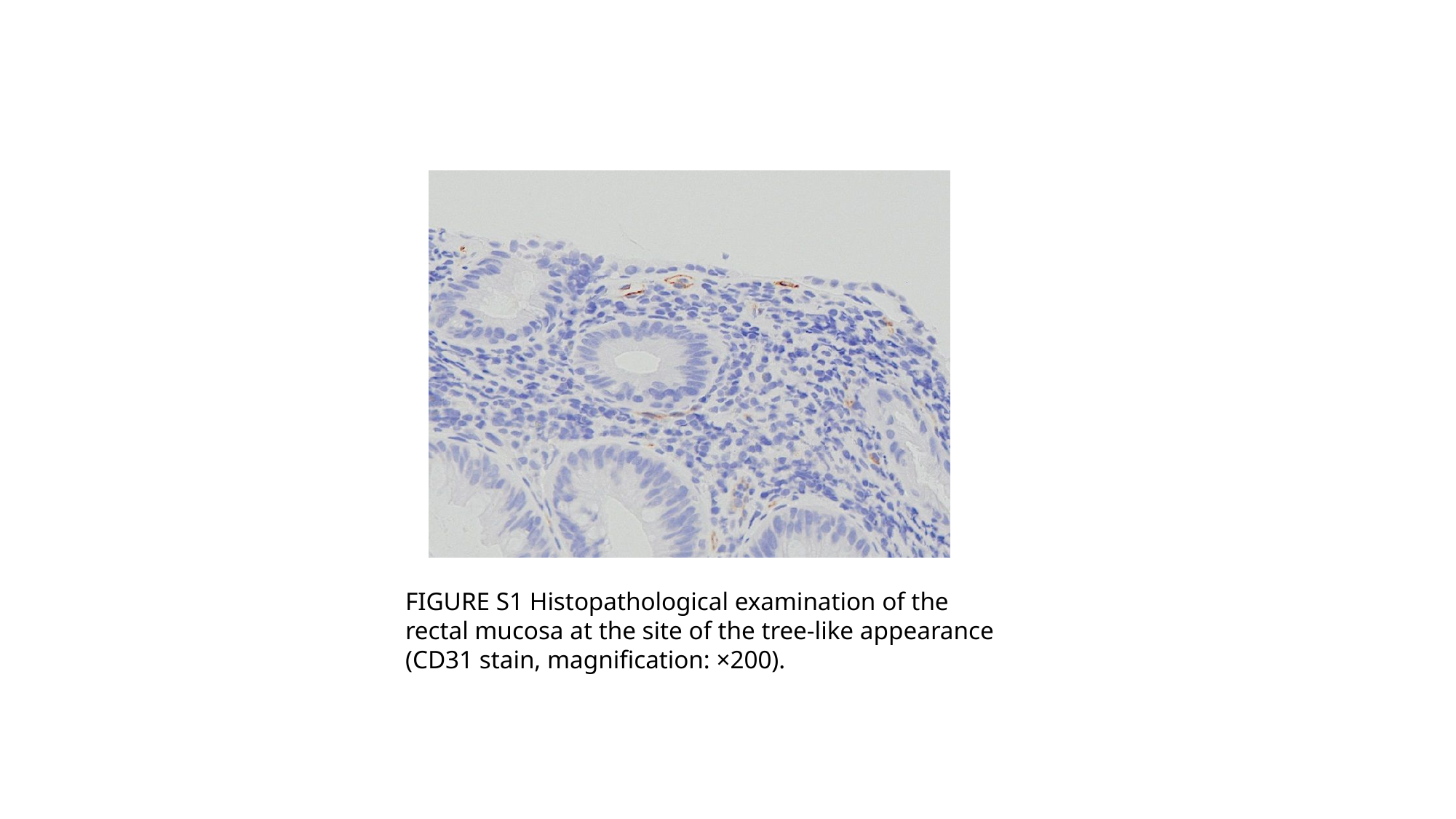

FIGURE S1 Histopathological examination of the rectal mucosa at the site of the tree-like appearance (CD31 stain, magnification: ×200).
